# Dialectics and Implications of Natural Neurotropic Autoantibodies in Neurological Disease and Rehabilitation

**DOI:** 10.1080/10446670410001722221

**Published:** 2004-06

**Authors:** A. B. Poletaev, A. A. Abrosimova, M. A. Sokolov, A. B. Gekht, V. V. Alferova, E. I. Gusev, T. Ya. Nikolaeva, C. Selmi

**Affiliations:** 1 Medical Research Center “Immunculus” Moscow Russia; 2 Department of Neurology Russian State Medical University Moscow Russia; 3 Yakutian State Research Center Yakutsk Russia; 4 Division of Rheumatology, Allergy, and Clinical Immunology University of California Davis CA USA

## Abstract

The role of natural idiotypic (Id-Abs) and anti-idiotypic (AId-Abs) autoantibodies against neuro-antigens observed in different neurological disorders is not fully understood. In particular, limited experimental evidence has been provided concerning the qualitative and quantitative serological response after acute injuries of the central nervous system or during chronic mental diseases. In this study, we analyzed the specific Id-Abs and AId-Abs serological reactivities against 4 neuro-antigens in a large population of patients with ischemic stroke, schizophrenia, as well as healthy individuals. Patients with ischemic stroke were tested at different time points following the acute stroke episode and a correlation was attempted between autoantibodies response and different patterns of functional recovery. Results showed variable and detectable Id-Abs and AId-Abs in different proportions of all three populations of subjects. Among patients with different functional recovery after ischemic stroke, a difference in time-related trends of Id-Abs and AId-Abs was encountered. Our observations suggest that changes in the production of natural neurotropic Abs may engender a positive homeostatic, beside a possible pathogenic effect, in specific neurological disorders.


*Clinical & Developmental Immunology*, June 2004, Vol. 11 (2), pp. 151–156

Taylor & Francis healthsciences

### Dialectics and Implications of Natural Neurotropic Autoantibodies in Neurological Disease and Rehabilitation

A.B. POLETAEV[a,*], A.A. ABROSIMOVA[a], M.A. SOKOLOV[a], A.B. GEKHT[b], V.V. ALFEROVA[b], E.I. GUSEV[b], T.YA. NIKOLAEVA[c] and C. SELMI[d]

[a]*Medical Research Center "Immunculus", 14 Otkrytoje Shosse 107370 Moscow, Russia;* [b]*Department of Neurology, Russian State Medical University, Moscow, Russia;* [c]*Yakutian State Research Center, Yakutsk, Russia;* [d]*Division of Rheumatology, Allergy, and Clinical Immunology, University of California, Davis, CA, USA*

The role of natural idiotypic (Id-Abs) and anti-idiotypic (AId-Abs) autoantibodies against neuro-antigens observed in different neurological disorders is not fully understood. In particular, limited experimental evidence has been provided concerning the qualitative and quantitative serological response after acute injuries of the central nervous system or during chronic mental diseases. In this study, we analyzed the specific Id-Abs and AId-Abs serological reactivities against 4 neuro-antigens in a large population of patients with ischemic stroke, schizophrenia, as well as healthy individuals. Patients with ischemic stroke were tested at different time points following the acute stroke episode and a correlation was attempted between autoantibodies response and different patterns of functional recovery. Results showed variable and detectable Id-Abs and AId-Abs in different proportions of all three populations of subjects. Among patients with different functional recovery after ischemic stroke, a difference in time-related trends of Id-Abs and AId-Abs was encountered. Our observations suggest that changes in the production of natural neurotropic Abs may engender a positive homeostatic, beside a possible pathogenic effect, in specific neurological disorders.

*Keywords*: Natural autoantibodies; Idiotypic/anti-idiotypic antibodies; Ischemic stroke; Schizophrenia

### INTRODUCTION

Almost a century ago, Vassily K. Khoroshko, a pioneer researcher at Moscow University, performed the first experiments of immunization of dogs using brain homogenates and studied the induced behavioral changes. His observations led to the hypothesis that neurotoxic antibodies could determine a number of mental and neurological disorders (Khoroshko, 1912). Since then, the links between neuropathology and immunopathology have been widely studied but still remain poorly understood. The reasons for this lack of progress are not clear, possibly involving incorrect interpretations (likely caused by excessive simplification) of previously obtained data or the skeptical disbelief of many scientists regarding available evidence. In this paper, we attempt to address the issue by illustrating our recent findings.

It is now accepted that the immune, nervous and endocrine functions play integrated roles within the human regulatory meta-system. Accordingly, disturbances of any of the three components eventually lead to compensatory changes in the activity of the others. As an example, shifts in serum natural autoantibodies (auto-Abs) against nervous cells proteins, such as neurotropic idiotypic auto-Abs (Id-Abs) described in mental and neurological diseases (Poletaev *et al.*, 2000, 2002), have been shown to be associated with shifts in anti-idiotypic auto-Abs (AId-Abs). It is still unknown, however, if changes in auto-Abs titers constitute a non-pathological physiological response to an injurious insult or whether they characterize the pathological process, possibly worsening the disorder. Since Dr Khoroshko's first findings, the presence of auto-Abs and increases in their titers have been commonly considered signs of disease. However, recent data showed the limits of such a "classical" position indicating that natural auto-Abs (including Id-Abs) can be physiological components of the immune system, involved mainly in the regulation of cell functions (Shoenfeld and Isenberg, 1992). For example, the temporary rise of Id-Abs production/titers following a neurological insult (such as a stroke) should be considered, at least in part, as a sign of recovery, rather than a marker of disease progression. It is still poorly understood, however, how individual immunological changes, such as raised auto-Abs, relate to pathological conditions and whether such changes may be considered qualitatively and quantitatively adequate. In the first part of the present study, in an attempt to evaluate

*Corresponding author. Tel.: +97-95-262-83-50. Fax: +97-95-475-37-80. E-mail: vtchernn@rol.ru

ISSN 1740-2522 print/ISSN 1740-2530 online © 2004 Taylor & Francis Ltd
DOI: 10.1080/10446670410001722221

the sensitivity of our methods, we compared the presence of specific Id-Abs and corresponding AId-Abs obtained from rabbits immunized with the neurotropic protein S100. We then extensively studied specific Id-Abs and AId-Abs against 4 neural antigens (Ags) in a large population of patients who suffered ischemic stroke (IS), as well as patients affected by schizophrenic disorder (SCH) and healthy subjects (HS) by using a new ELISA protocol. A serological analysis of patients with IS was also performed at different time points after the acute episode and a correlation between auto-Abs response and different patterns of functional recovery was attempted.

### MATERIALS AND METHODS

### Immunization of Rabbits

Cytoplasmic brain protein S100, cytoskeletal glial fibrillary acidic protein (GFAP), and 65-kDa brain cell membrane protein MP65 were purified from bovine brain as previously described (Poletaev *et al.*, 2000). Nerve growth factor (NGF) was purchased from Sigma–Aldrich (Moscow, Russia). Adult female rabbits were injected subcutaneously with 200 $\mu$g of Ag biweekly for 6 weeks. The Ags were suspended in 500 $\mu$l of 0.14 M NaCl and carefully mixed with equal volume of Complete Freund Adjuvant (Difco, Kansas City, MO, USA). Blood samples were collected 1 week after the final injection and sera was obtained by centrifugation.

### Evaluation of Anti-idiotypic Fab-fragments or Native Ag for ELISA

The detection of Abs against native Ag or Ag-mimicking AId-Fab-fragments was investigated through the evaluation of S100 and S100-mimicking AId-Fab-fragments by two different approaches.

First, 96-well immunoplates (Nunc maxisorp, Denmark) were coated with either native S100 protein, S100-mimicking AId-Fab-fragments obtained from rabbit hyperimmune serum, bovine serum albumin (BSA) or Fab-fragments of non-immune rabbit IgG (all at 3 ug/ml concentration in 0.1 M carbonate buffer pH 9.5) overnight at 4°C. Plates were washed three times using PBS with 0.05% Tween (PBST), and 100 ul of serum from immunized rabbits (at serial dilutions) were added to each well for 16 h at 4°C. *O*-phenylenediamin/$H_2O_2$ as substrate was then added and the optical density (OD) measured at 490 nm. Second, 96-well immunoplates (Nunc maxisorp) were coated with S100-mimicking AId-Fab-fragments obtained from rabbit hyperimmune serum as previously described and washed. To each well was then added either S100 protein (10 ug) or BSA (10 ug). The plates were gently shaken for 3 h at 37°C, washed, and added with increasing dilutions (from 1:100 to 1:12,800) of anti-S100 rabbit antiserum. Plates were then analyzed as described above, after the addition of substrate.

### Enzyme Immunoassay (EIA) Test-System ELI-N-Test

EIA was performed as previously described (Poletaev *et al.*, 2003). S100, GFAP, MP-65 and NGF were used for rabbits immunization as well as for coupling with CNBr-Sepharose (Pharmacia Biotech, Uppsala, Sweden) for affinity purification of serum polyclonal idiotypic Abs (Id-Abs) against these Ags. Id-Abs were additionally purified upon HiTrap Protein-G (Pharmacia Biotech), and their Fab-fragments (Fab-S100, Fab-GFAP, Fab-MP65, Fab-NGF) were obtained after pepsin hydrolysis. The specific Fab-fragments obtained were immobilized upon CNBr-Sepharose and used for separation of anti-idiotypic Abs (AId-Abs) from the same portions of antisera which were used to obtain Id-Abs. AId-Abs against idiotypic Fab-S100, Fab-GFAP, Fab-MP65, Fab-NGF were purified upon HiTrap Protein-G, passed through columns with immobilized non-immune rabbit IgG, and Fab-fragments of corresponding AId-Abs were prepared. Id-Fab-fragments against S100, GFAP, MP65 and NGF, and corresponding AId-Fab-fragments were used as the main components of the test-system ELI-N-Test for detection of appropriate AId-Abs and Id-Abs in serum samples.

### Evaluation of Serum Immunoreactivity (IR)

Study of IR EIA was performed as previously described (Poletaev *et al.*, 2000, 2003). Briefly, serum samples were diluted 1:200 in 0.05% Tween 20 PBS (PBST), and 96-well immunoplates (Nunc maxisorp) were coated with Id- or AId-Fab-fragments. ELISA protocol followed previous description (Poletaev *et al.*, 2003) and IR of each sample was calculated in conditional units (CU) (Shoenfeld and Isenberg, 1992), using the following formula:

$$\mathrm{IR} = \frac{R - x(\mathrm{Fab}) \times 100}{R - \mathrm{st}(\mathrm{Fab})} - 100$$

where $R - x(\mathrm{Fab})$ is the EIA OD of patients' sera and $R - \mathrm{st}(\mathrm{Fab})$ the OD of a known control serum. A highly standardized sample of human immunoglobulins of IgG class was used as control sample and evaluated in each immunoplate in parallel with analyzed serum samples.

The Id–AId reactions ratio for each serum sample was calculated as described (Poletaev *et al.*, 2000, 2003) by the following equation:

$$\frac{(\mathrm{CU} + 100)_{\text{reaction with anti-idiotpic Fab}}}{(\mathrm{CU} + 100)_{\text{reaction with corresponding idiotpic Fab}}}$$

Normal values were considered between −35 and 40 CU and between 0.7 and 1.3, respectively, as previously described (Poletaev *et al.*, 2000, 2002).

### Subjects

We obtained clinical information and serum samples from 109 patients with recent history of cortical or sub cortical IS, 38 patients with a diagnosis of SCH and 220 HS.

Patients with IS (63 females, age 65 ± 4 years) were enrolled among inpatients admitted at the Neurology Department of the Russian State Medical University. Based on functional recovery after the stroke and Barthel Index (Mahoney and Barthel, 1965), patients were subdivided in 4 groups: Group 1 ($n = 36$) included patients with good functional recovery and Barthel score between 100 and 85, Group 2 ($n = 35$) patients with satisfactory recovery and Barthel score between 80 and 65, Group 3 ($n = 27$) patients with unsatisfactory recovery and Barthel score between 60 and 45 balls, and Group 4 ($n = 11$) patients deceased within 12 days from the IS episode. Serum samples were obtained at 3–5, 10–14 and 21–35 days after stroke. Moreover, serum samples at 6–8 months were also obtained from 12 patients from Group 3 and 10 patients from Group 1.

Subjects affected by SCH (22 females, age 42 ± 9 years) were enrolled among inpatients admitted at the Karelian Republican Psychiatry Hospital (Director Dr V.M. Zlunikin) for SCH of paranoid form during the acute period and the diagnosis was based upon MMPI criteria (Walters, 1983). Disease duration in this group of patients ranged between 2 months and 5 years. Serum samples were obtained before any acute medical treatment was started.

HS (155 females, age 36 ± 6 years) included individuals without signs or symptoms of allergy, current infection, or endocrine, mental or neurological disorders.

### Statistical Analysis

The chi-square test and Mann–Whitney U-test were used for the comparison of categorical and continuous variables, respectively. All analysis were two-tailed and a $P$ value $<0.05$ was considered statistically significant.

### RESULTS

Following rabbit immunization with S100 we assessed the presence in sera of auto-Abs against S100 (Id-Abs), an S100-mimicking Fab fragment (AId-Abs), BSA, and a control IgG Fab fragment at serial dilutions (Table I(A)). Id-Abs and AId-Abs titers were found to be significantly higher than what observed against control IgG at dilutions as high as 1:800 for Id-Abs and 1:6400 for AId-Abs (Table I(A)). Further, the absorption of sera with S100 but not with BSA significantly reduced AId-Abs reactivity observed at all dilutions (Table I(B)).

The presence of Id-Abs and AId-Abs against S100, GFAP, MP65 and NGF was investigated in sera obtained from patients with IS ($n = 109$) or SCH ($n = 38$), as well as HS ($n = 220$). Results were expressed in two different forms: the absolute values obtained for Id-Abs or AId-Abs reactivities (expressed in CU) and the ratio between such values were analyzed.

Id-Abs and AId-Abs reactivities observed among HS are shown in Table II. This group (Table II(A)) presented similar IR values for Id-Abs and AId-Abs, as reflected by Id/AId ratios within the normal range (1.1 ± 0.2 for S100; 1.2 ± 0.1 for GFAP; 1.0 ± 0.1 for MP65; 1.1 ± 0.1 for NGF). However, when the percentage of subjects presenting IR values outside the normal range was examined (Table II(B)), a higher fraction of subjects presented altered AId-Abs values, compared with Id-Abs, against MP65 and NGF (5 vs. 1.8% and 5.7 vs. 3.5%, respectively; $P =$ NS).

Among patients with SCH, normal values of IR against the 4 Ags were encountered in 36–51% of patients, with similar values for Id-Abs and AId-Abs and the most frequent alterations encountered against GFAP (Table III(A)). The Id-Abs/AId-Abs ratio was found to be within normal range in only 29–57% of subjects, with major alterations observed against GFAP (65% presented a ratio below normal range) (Table III(B)). Altered values of IR (i.e. outside the defined normal range) against the 4 Ags were found more frequently among patients with SCH compared to healthy subjects (49–64% in SCH vs. 1.8–5.7% in HS; $P < 0.05$). Similar results were obtained for frequencies of abnormal Id-Abs/AId-Abs ratios (46–74% in SCH vs. 2–8% in HS; $P < 0.05$). Combined results showed that alterations in IR against any of the 4 Ags were found in 97% of patients with SCH (vs. 6% among HS; $P < 0.05$) while combined alterations in Id-Abs/AId-Abs ratio were also detected in 89.5% of patients with SCH (vs. 11% among HS; $P < 0.05$).

TABLE I (A) IR of anti-S100 rabbit antiserum at increasing dilutions against S100, S100-mimicrying Fab-fragments of anti-idiotypic Abs, BSA and Fab-fragments of non-immune IgG; (B) IR of anti-S100 rabbit antiserum at increasing dilutions against S100-mimicrying Fab-fragments of anti-idiotypic Abs after adsorption with S100 and BSA (Values are expressed as mean ± standard deviation OD)

| | (A) Antigen | | | | (B) Adsorbing agent | |
|---|---|---|---|---|---|---|
| Antiserum dilution | S100 | AId-Fab S100 | BSA | Control IgG Fab | S100 | BSA |
| 1:100 | 1.12 ± 0.20 | 2.15 ± 0.30 | 0.20 ± 0.14 | 0.21 ± 0.08 | 0.29 ± 0.12 | 0.95 ± 0.16 |
| 1:200 | 0.73 ± 0.11 | 1.88 ± 0.15 | 0.12 ± 0.11 | 0.22 ± 0.09 | 0.19 ± 0.09 | 0.80 ± 0.11 |
| 1:400 | 0.51 ± 0.12 | 1.53 ± 0.15 | 0.17 ± 0.13 | 0.19 ± 0.11 | 0.21 ± 0.10 | 0.62 ± 0.14 |
| 1:800 | 0.33 ± 0.08 | 0.98 ± 0.17 | 0.14 ± 0.08 | 0.14 ± 0.14 | 0.18 ± 0.12 | 0.41 ± 0.10 |
| 1:1.600 | 0.16 ± 0.10 | 0.72 ± 0.09 | 0.13 ± 0.11 | 0.14 ± 0.12 | 0.24 ± 0.16 | 0.21 ± 0.13 |
| 1:3.200 | 0.11 ± 0.09 | 0.41 ± 0.11 | 0.14 ± 0.12 | 0.15 ± 0.07 | 0.27 ± 0.14 | 0.19 ± 0.18 |
| 1:6.400 | 0.14 ± 0.15 | 0.21 ± 0.12 | 0.12 ± 0.12 | 0.16 ± 0.09 | 0.25 ± 0.13 | 0.17 ± 0.14 |
| 1:12.800 | 0.12 ± 0.12 | 0.16 ± 0.14 | 0.17 ± 0.07 | 0.12 ± 0.11 | 0.28 ± 0.15 | 0.15 ± 0.16 |

TABLE II (A) Serum IR of 220 healthy individuals against Id and AId antigens. Reactivities are expressed in OD values (mean ± standard deviation) and Id-Abs / AId-Abs ratios; (B) Percentages of healthy subjects with abnormal IR values (normal values are considered between −35 and 40 CU for IR, and between 0.7 and 1.3 for ratio)

| (A) | Id-Abs | AId-Abs | Id-Abs / AId-Abs ratio |
|---|---|---|---|
| S100 | 2.2 ± 0.3 | 1.8 ± 0.4 | 1.1 ± 0.2 |
| GFAP | 6.4 ± 0.6 | 6.0 ± 0.7 | 1.2 ± 0.1 |
| MP65 | 1.2 ± 0.5 | 0.8 ± 0.2 | 1.0 ± 0.1 |
| NGF | 0.2 ± 0.4 | 0.3 ± 0.2 | 1.1 ± 0.1 |
| (B) | Id-Abs < −35 or > 40 CU (%) | AId-Abs < −35 or > 40 CU (%) | Id-Abs / AId-Abs ratio < 0.7 or > 1.3 (%) |
| S100 | 4.1 | 3.7 | 3 |
| GFAP | 4.1 | 4.8 | 8 |
| MP65 | 1.8 | 5 | 2 |
| NGF | 3.5 | 5.7 | 7 |

Alterations in IR (regarding both Id-Abs and AId-Abs) were found in all 109 patients with IS, regardless of their functional outcome against S100, GFAP and MP65 at all examined time-points ($P < 0.01$ vs. HS for all Ags). On the other hand, isolated alterations of Id-Abs/AId-Abs ratios were encountered in 16% of patients with IS (vs. 11% among HS; $P =$ NS). In the subgroup of patients with IS resulting in post-stroke dementia examined after 6–8 months (previously included in Group 3), an increase in Id-Abs/AId-Abs ratio was encountered in 6 cases (vs. 4 with decreased ratio; $P =$ NS).

Patients with IS were stratified in 4 groups according to their functional outcome. At 3–5 days after IS, patients in Groups 1 and 2 (better recovery) presented lower anti-S100 Id-Abs compared to patients in Groups 3 and 4 (worse recovery) [IR median 27 CU (range −26, +84) vs. 44 CU (−5, +156); $P < 0.05$], while showing higher reactivities at 10–14 days [IR 36 CU (−10, +107) vs. 10 CU (−54, +46); $P < 0.02$] (Fig. 1). Similar data were obtained for IR against other investigated Ids-Abs and AIds-Abs (data not shown).

TABLE III Fractions of patients with SCH presenting altered IR against different antigens, expressed as CU values of Id-Abs and AId-Abs (A) and Id-Abs / AId-Abs ratio (B) against each Ag

| (A) | IR (CU) | Id-Abs | AId-Abs |
|---|---|---|---|
| S100 | < −35 | 22% | 27% |
| | −35, 40 range | 49% | 42% |
| | > 40 | 29% | 31% |
| GFAP | < −35 | 25% | 12% |
| | −35, 40 range | 46% | 36% |
| | > 40 | 29% | 48% |
| MP65 | < −35 | 7% | 31% |
| | −35, 40 range | 47% | 42% |
| | > 40 | 46% | 27% |
| NGF | < −35 | 28% | 21% |
| | −35, 40 range | 51% | 48% |
| | > 40 | 21% | 31% |
| (B) | Id-Abs/AId-Abs ratio | | |
| S100 | < 0.7 | 25% | |
| | 0.7, 1.3 range | 54% | |
| | > 1.3 | 21% | |
| GFAP | < 0.7 | 65% | |
| | 0.7, 1.3 range | 29% | |
| | > 1.3 | 5% | |
| MP65 | < 0.7 | 33% | |
| | 0.7, 1.3 range | 57% | |
| | > 1.3 | 9% | |
| NGF | < 0.7 | 20% | |
| | 0.7, 1.3 range | 53% | |
| | > 1.3 | 26% | |

TABLE IV Id-Abs/Aid-Abs ratios in patients with good rehabilitation after stroke and in patients with post-stroke dementia

| | Id-Abs/Aid-Abs (S100) | Id-Abs/Aid-Abs (GFAP) | Id-Abs/Aid-Abs (MP65) | Id-Abs/Aid-Abs (NGF) |
|---|---|---|---|---|
| Patients with post-stroke dementia | | | | |
| 1 | 1.1 | 1.0 | 1.0 | 1.0 |
| 2 | **0.6** | **1.6** | 1.0 | 0.9 |
| 3 | **2.2** | 1.0 | **4.0** | 0.7 |
| 4 | 1.0 | 0.9 | 1.0 | **1.4** |
| 5 | 1.2 | 0.8 | 0.7 | 1.1 |
| 6 | 1.0 | **0.5** | 0.8 | 1.0 |
| 7 | 0.8 | 0.9 | 0.9 | 0.9 |
| 8 | 0.9 | 0.9 | 0.9 | 0.9 |
| 9 | **0.5** | 0.9 | 0.9 | 1.3 |
| 10 | **1.4** | 0.7 | 0.9 | 0.9 |
| 11 | **0.6** | 0.8 | 1.3 | 0.9 |
| 12 | **1.4** | 1.0 | 1.0 | 1.1 |
| Patients with good post-stroke rehabilitation | | | | |
| 1 | 1.2 | 1.1 | 1.3 | 1.0 |
| 2 | 0.9 | 1.0 | 0.8 | 1.0 |
| 3 | 1.1 | 1.2 | 1.1 | 1.0 |
| 4 | 0.8 | 1.0 | 0.9 | 0.8 |
| 5 | 1.1 | 0.9 | 1.0 | 0.9 |
| 6 | 0.9 | 1.1 | 1.0 | 1.1 |
| 7 | 1.2 | 1.0 | 1.3 | 1.2 |
| 8 | 1.0 | 1.2 | 1.1 | 0.8 |
| 9 | 1.1 | 1.0 | 1.2 | 0.7 |
| 10 | 1.1 | 1.2 | 1.0 | 1.2 |

Abnormal data are indicated by bold.

### DISCUSSION

The final stages of Ag processing *in vivo* are accompanied by the presentation of different epitopes which are derived from the processing of specific proteins, as complexes with major histocompatibility complex molecules. These events lead to the production of several polyclonal Abs, some of which unable to recognize the native Ag form, largely due to sterical impediments (Poletaev *et al.*, 2003). Such phenomenon often leads to a reduced sensitivity of the commonly used laboratory methods for the detection of polyclonal Abs. Therefore, as Fab-fragments of AId-Abs may mimic different Ag epitopes (Poletaev *et al.*, 2002, 2003), using such fragments instead of native Ag may increase the sensitivity of immunological methods. The results described herein obtained testing antiserum of rabbits immunized S100 or the corresponding AId-Fab-fragments seem to confirm such hypothesis (Table I). Sera from immunized rabbits presented stronger reactivity against S100-mimicking AId-Fab-fragments than against S100 protein (Table I(A)). Moreover, no reactivity was

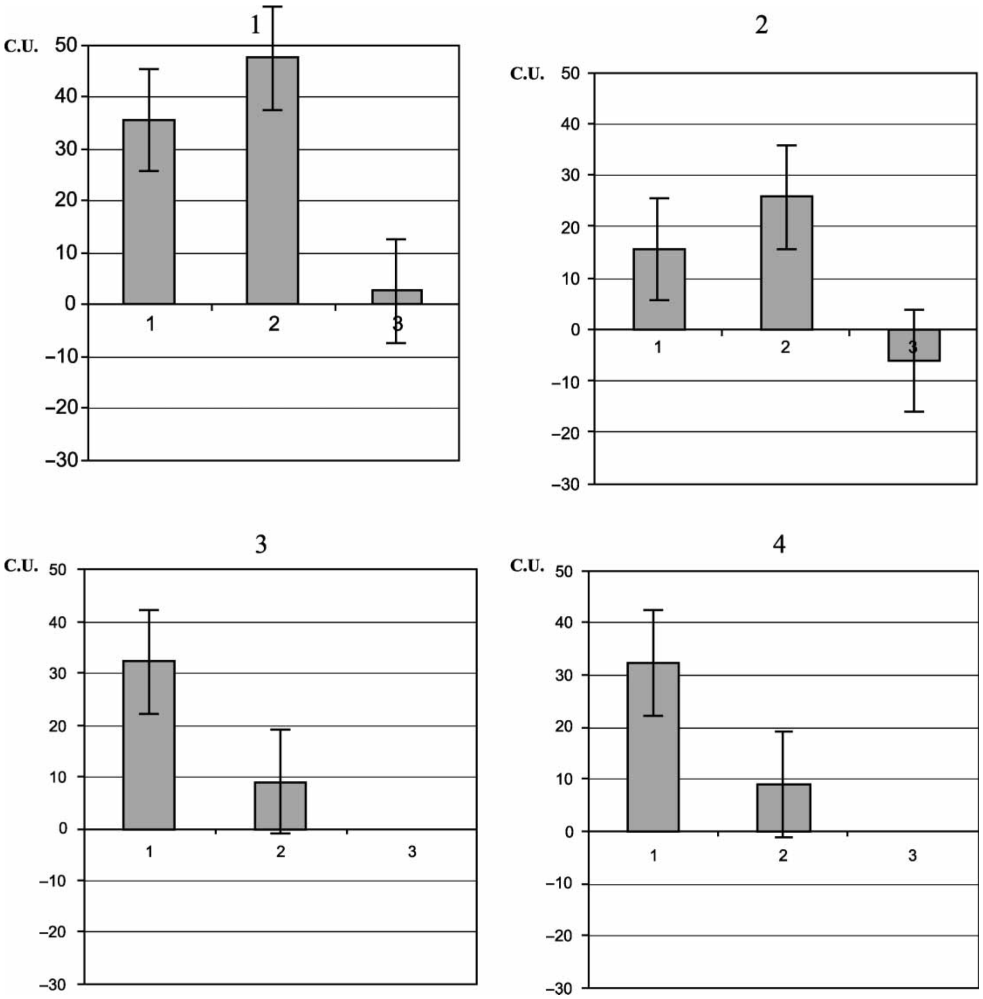

FIGURE 1 Dynamic changes over time in serum IR (expressed in CU) of anti-S100 Id-Abs in patients with IS subdivided in 4 groups according to the functional recovery (see text). Evaluations of IR were executed at 3–5 (column 1), 10–14 (column 2) and 21–35 (column 3) days after the IS episode.

observed against BSA or IgG Fab-fragments from control rabbits (Table I(A)). Specificity of Abs binding was then proven by the inhibition of reactivities observed when S100, but not BSA, were added (Table I(B)). These observations, therefore, were used to develop an Id-AId-based test-system ELI-N-Test to detect neurotropic Id-Abs as well as their AId counterparts in human serum samples (Russian Patent in preparation; Certificate of priority #2003131816, 30 October 2003).

A confirmation that neurotropic auto-Abs may play a physiological role was possibly found in the detectable presence of Id-Abs against all investigated proteins as well as AId-Abs (i.e. constitutively expressed natural neurotropic Abs) in sera from all investigated healthy subjects (HS group; Table II). For practical purposes, we estimated the normal ranges for IR (in CU—see above) for each antigen used and the ratio between CUs for each pairs of Id-Abs and AId-Abs (between −35 and 40 CU and between 0.7 and 1.3, respectively). Our findings showed that IR and ratio were within these ranges in over 95% of HS and did not correlate with age or sex of the individuals (data not shown), thus confirming previous reports (Poletaev *et al.*, 2000, 2002). These observations seem to indicate that optimal (physiological) levels of different neurotropic auto-Abs are maintained within narrow limits by powerful feedback mechanisms, thus suggesting a role for these molecules in regulatory mechanisms.

A very different scenario was encountered among patients with neurological disorders. In 97% of SCH and 100% of IS patients, in fact, abnormal changes of IR could be detected against all or some of the investigated Ags. We note that, for SCH, our results obtained with GFAP appear to be compatible with previous reports indicating an increased expression of this protein specific for the disease (Johnston-Wilson *et al.*, 2000). Interestingly, dynamic changes were observed across serial sampling among patients attributed to different groups according to the functional outcome of the IS (Fig. 1). In all 4 groups, an early elevation followed by a significant decrease over time was observed in anti-S100 Id-Abs levels. Similar trends were also observed for other Id-Abs as well as for all AId-Abs (data not shown). Different patterns of changes in Id-Abs over time, however, were found among patients with different outcomes. For example, patients with better functional recovery after IS (Groups 1 and 2) presented the highest levels of IR after 10–14 days from the IS episode, while subjects with worse recovery (Groups 3 and 4) presented an earlier peak (3–5 days) with subsequent significant decrease over time.

Our results once again raise the question if the observed changes should only be regarded as signs of disease, as commonly thought. Available evidence seems to suggest the opposite, thus implying a possible compensatory nature of the observed changes. A further question regards the adequacy (in location, magnitude and timing) of such reaction. The observations reported herein point towards a possible role for Id-Abs in the physiological attempt performed by the general network of constitutively expressed natural a-Abs, named "Immunculus", (Poletaev and Osipenko, 2003) (which may be roughly defined as the internal "mould" of the optimal metabolic state as reflected by the immune system), to compensate for the neurological loss and to re-establish the pre-existing balance, as indicated by the changes in "failure" scenarios. Interestingly, however, data obtained in the subgroup of patients in which serum was analyzed also at 6–8 months after IS, representing the two extremities of the range of functional recovery (previously represented by Groups 1 and 3), showed the presence of an abnormal Id-Abs/AId-Abs ratio in a higher percentage among patients with poor recovery resulting in post-stroke dementia (Table IV). This observation could indicate a possible change in immunospecificity of the IR, hence showing also a qualitative shift accompanying more severe outcomes. Interestingly, a similar alteration pattern in specificity ratios was encountered in the vast majority of patients with SCH (89.5%). In this population, moreover, other peculiarities in neurotropic auto-Abs were found, with isolated alterations, rather than generalized (as seen in IS), found in 73% of sera. No specific correlation between clinical features and Ag-specific neurotropic auto-Abs patterns was found (data not shown), although the cross-sectional nature of this part of the study should be taken into account as a possible reason for such absence of correlation.

Further, the findings described herein seem to indicate a possible correlation between the adequacy of changes in the neurotropic auto-Abs and the pathophysiology of neurological disorders such as SCH or the different degrees of functional recovery after IS. Such data also seem to be in accordance with what shown during long follow-up on newborns from mothers who suffered massive herpetic and cytomegalovirus infection during pregnancy (Poletaev *et al.*, 2002). In 12–15% of such cases newborns presented abnormal (either elevated or prominently depressed) serum contents of different neurotropic auto-Abs during several years after birth, and more than 70% of them developed neurological or mental problems. In particular, we showed a different longitudinal pattern of serum reactivity in patients with different functional recovery. We could once more assume, therefore, that the different occurrence over time of the peak neurotropic auto-Abs titers observed in patients with good clinical recovery after stroke may represent an adaptive reaction of the immune system aimed to the compensation of the neurological insult and the resulting rehabilitation. The mechanisms leading to this phenomenon are poorly understood, but we can suggest a role for the cell growth stimulation exerted by a number of neurotropic auto-Abs (Shoenfeld and Isenberg, 1992; Zaichik and Churilov, 2001; Asakura *et al.*, 1996).

In conclusion, we suggest that changes in production of neurotropic auto-Abs may play a positive homeostatic role in defined cases, as well as possibly be pathognomonic, in different neurological disorders, similarly to what observed for other biologically active regulatory molecules. It is impossible, with the present state of knowledge, to rule out the possibility that neurotropic auto-Abs might be mere epiphenomenon of other events. It seems clear, however, that a better understanding of the dialectics of neurotropic auto-Abs, now often regarded as a theoretical matter, can potentially provide better tools in the diagnosis and clinical management of neurological and mental disorders and possibly indicate targets for new treatments. Only overtaking the skepticism raised by these and previous experimental data by a part of the scientific community will allow a more objective and productive evaluation of this important issue.

### *References*

Asakura, K., Pogulis, R.J., Pease, L.R. and Rodriguez, M. (1996) "A monoclonal autoantibody which promotes central nervous system remyelination is highly polyreactive to multiple known and novel antigens", *J. Neuroimmunol.* **65**, 11–19.

Johnston-Wilson, N.L., Sims, C.D., Hofmann, J.P., *et al.* (2000) "Disease-specific alterations in frontal cortex brain proteins in schizophrenia, bipolar disorder, and major depressive disorder. The Stanley Neuropathology Consortium", *Mol. Psychiatr.* **5**, 142–149.

Khoroshko, V.K. (1912) Reaction of the Living Organism after Administration of the Nervous Tissue, Doctoral Thesis (Moscow University Publishing, Moscow).

Mahoney, F.I. and Barthel, D.W. (1965) "Functional Evaluation: The Barthel Index", *Md. State Med. J.* **14**, 61–65.

Poletaev, A. and Osipenko, L. (2003) "General network of natural autoantibodies as immunological homunculus (Immunculus)", *Autoimmun. Rev.* **2**, 264–271.

Poletaev, A.B., Morozov, S.G., Gnedenko, B.B., Zlunikin, V.M. and Korzhenevskey, D.A. (2000) "Serum anti-S100b, anti-GFAP and anti-NGF autoantibodies of IgG class in healthy persons and patients with mental and neurological disorders", *Autoimmunity* **32**, 33–38.

Poletaev, A.B., Morozov, S.G. and Kovaliov, I.E. (2002) Regulatory Meta-system (Immuno-neuroendocrine Regulation of General Homeostasis) (Medicina, Moscow).

Poletaev, A.E., Alferova, V.V., Abrosimova, A.A., Kommissarova, I.A., Sokolov, M.A. and Gusev, E.I. (2003) "Natural neurotropic antibodies and pathology of the nervous system", *J. Neuroimmunol.* **1**, 11–17.

Shoenfeld, Y. and Isenberg, D.A. (1992) Natural autoantibodies: their physiological role and regulatory significance (CRC Press, Boca Raton, FL).

Walters, G.D. (1983) "The MMPI and schizophrenia: a review", *Schizophr. Bull.* **9**, 226–246.

Zaichik, A.S. and Churilov, L.P. (2001) General Pathophysiology (ELBI-SPb, St. Petersbourg).

